# Case report: Eisenmenger syndrome in a dog with ventricular septal defect: long term management and complications

**DOI:** 10.3389/fvets.2024.1393919

**Published:** 2024-11-01

**Authors:** Barbara Bruno, Paolo Savarino, Claudio Bussadori, Andrea Degiovanni, Elena Lardone, Alessia Bertero, Alberto Tarducci

**Affiliations:** ^1^Department of Veterinary Science, University of Turin, Grugliasco, TO, Italy; ^2^Clinica Veterinaria Gran Sasso, Milan, Italy

**Keywords:** congenital heart malformation, right-to-left shunting, sildenafil citrate, polycythemia, dog

## Abstract

A 9-month-old female Jack Russell Terrier was referred because of two episodes of suspected syncope. Echocardiography revealed a large, isolated mid-muscular ventricular septal defect (7 mm) with bidirectional shunting. A diagnosis of Eisenmenger syndrome and erythrocytosis (68.5% of hematocrit) was made and sildenafil therapy (0.5 mg/Kg twice daily) was initiated. Regular follow-up examinations were performed and Sildenafil therapy was gradually increased based on changes in clinical signs, packed cell volume and heart rate. The dog's clinical conditions have been stable without the need of phlebotomy for 4 years and 6 months, until April 2021 (month 74) (about 7 years of survival) when the dog had 80% of hematocrit and signs of hyperviscosity syndrome. The dosage of administered sildenafil reached gradually 6 mg/Kg DIE (2 mg/Kg three times a daily) in June 2022. Lastly, in November 2023 a closed pyometra was diagnosed and the dog was hospitalized and operated. The major concerns in the peri-operative period were hypotension and the profound hypoxemia, but the dog was discharged and recovered normal activity in approximately 11 days. This report describes the long-term management with sildenafil citrate of Eisenmenger syndrome secondary to a muscular ventricular septal defect with right-to-left shunting in a dog. Sildenafil therapy was always well tolerated with no side effects reported, even at higher dosage and drastically reduced the need for phlebotomy.

## Introduction

Ventricular septal defect (VSD) is a common congenital heart disease in dogs, reported particularly in breeds such as English Bulldog, English Springer Spaniel, and West Highland White Terrier (1). Ventricular defects can arise in any part of the interventricular septum but most frequently involve the membranous portion, whereas muscular apical or midventricular septal defects are rarely detected in small animals (1, 2). Left heart pressure is usually found to be higher than that in the right heart, with left-to-right shunting, but bidirectional or pulmonary-to-systemic shunting may occur across large septal defects and when right ventricular pressure is high (e.g. pulmonary stenosis or pulmonary arterial hypertension) (1). Eisenmenger syndrome (ES) indicates reversal of the shunt, and cyanotic heart disease usually develops before the age of 6 months (1, 3). In the literature, VSD in dogs accounts for 4.8% to 12.3%, but reversed shunt is less frequent, as reported in a retrospective case series, in which only 1 of 26 dogs affected by VSD had ES secondary to large VSD (7.4 mm diameter) (4–10).

Surgical correction of the defect is contraindicated in ES because the septal defect allows partial relief of the right ventricular overload and maintains left ventricular output. The best therapeutic option is medical management focused on supportive treatment (1, 11, 12). Erythrocytosis can exaggerate clinical signs associated with hypoxemia such as exercise intolerance, exertional dyspnea and syncope (13). Therapy aims to reduce blood viscosity by repeated phlebotomy or with myelosuppressive drugs (i.e., hydroxyurea), and decrease pulmonary hypertension (PH) with the administration of phosphodiesterase type-5 inhibitors (sildenafil citrate) or endothelin receptor antagonists (14–17). Three case series and a retrospective multicenter study have described different treatments for reversed patent ductus arteriosus (PDA) in dogs (13, 18–20). Côté et al. performed phlebotomy to control erythrocytosis as part of long-term management in 3 dogs, Moore et al. used chronic hydroxyurea therapy in 4 dogs, and Nakamura et al. found that administration of sildenafil over a 3-months period improved clinical signs and erythrocytosis in 5 dogs (18–20). In the retrospective study it was observed that dogs treated with sildenafil, at presentation, lived longer than those without treatment (13).

Here we have reported the management of dog with ES secondary to a muscular VSD with bidirectional shunting for more than 9 years. Last year the dog developed a pyometra and we have also described its intra and postoperative management.

## Case report

In January 2015, a 9-month-old female Jack Russell Terrier (body weight 4.5 Kg) was referred by a veterinary practitioner because of 2 episodes of suspected syncope that had occurred 1 month earlier, with loss of consciousness during mild physical activity in both instances. At presentation, cardiovascular examination revealed a heart rate (HR) of 160 bpm, cyanotic mucous membranes (MM), no heart murmur, normal and regular femoral and metatarsal arterial pulses, and presence of the hepatojugular reflux was observed. Systolic blood pressure was 120 mm Hg (Doppler Vet BP, Mano Médical).

**Figure 1:**
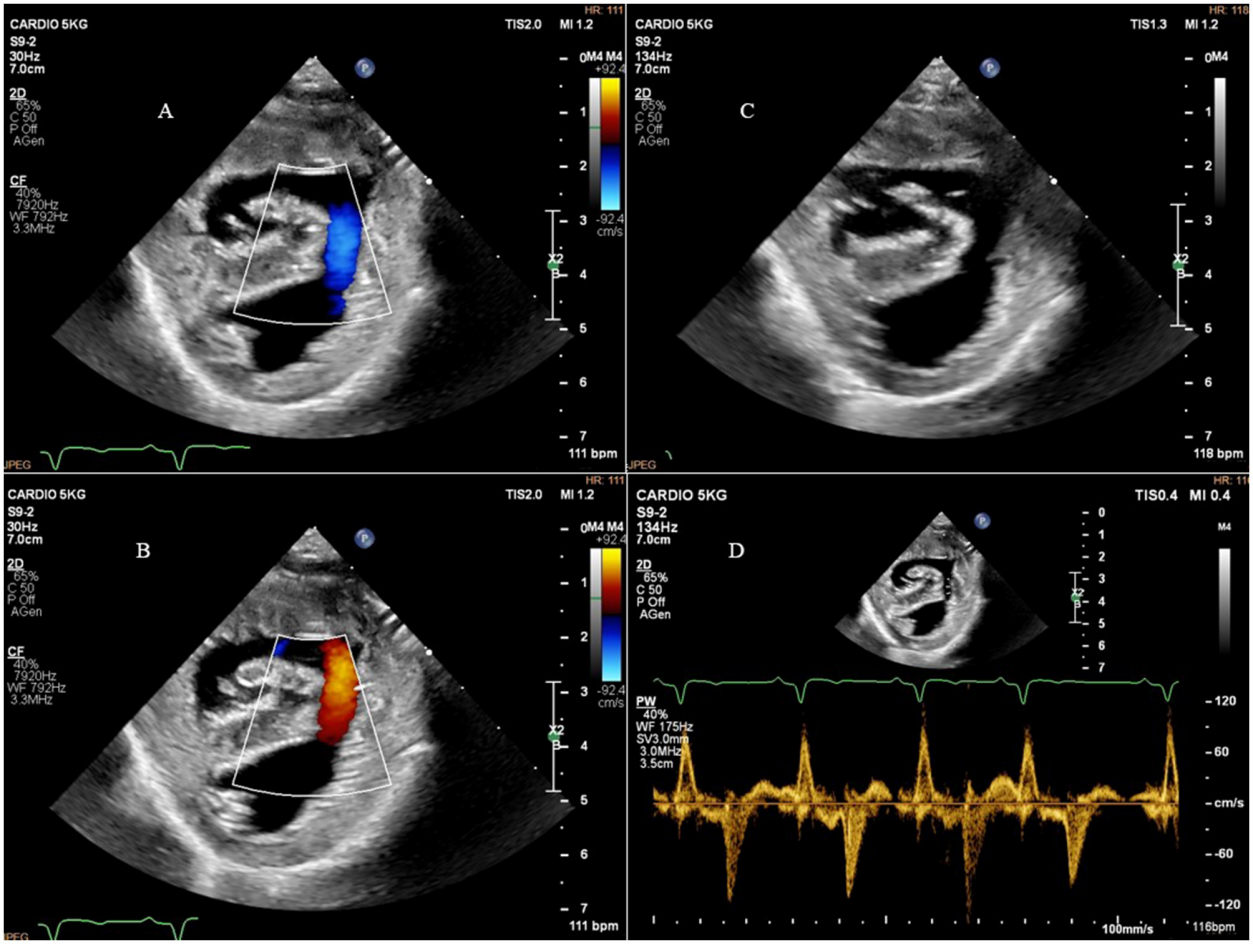
Right parasternal short axis view showing the ventricular septal defect and bidirectional shunting. **(A)** Right parasternal short axis view and color-flow Doppler of the bidirectional shunting through the ventricular septal defect during diastole; **(B)**, right parasternal short axis view and color-flow Doppler of the bidirectional shunting through the ventricular septal defect during systole; **(C)**, right parasternal short axis view showing the ventricular septal defect; **(D)**, right parasternal short axis view and pulsed-flow Doppler of the bidirectional shunting through the ventricular septal defect.

**Figure 2:**
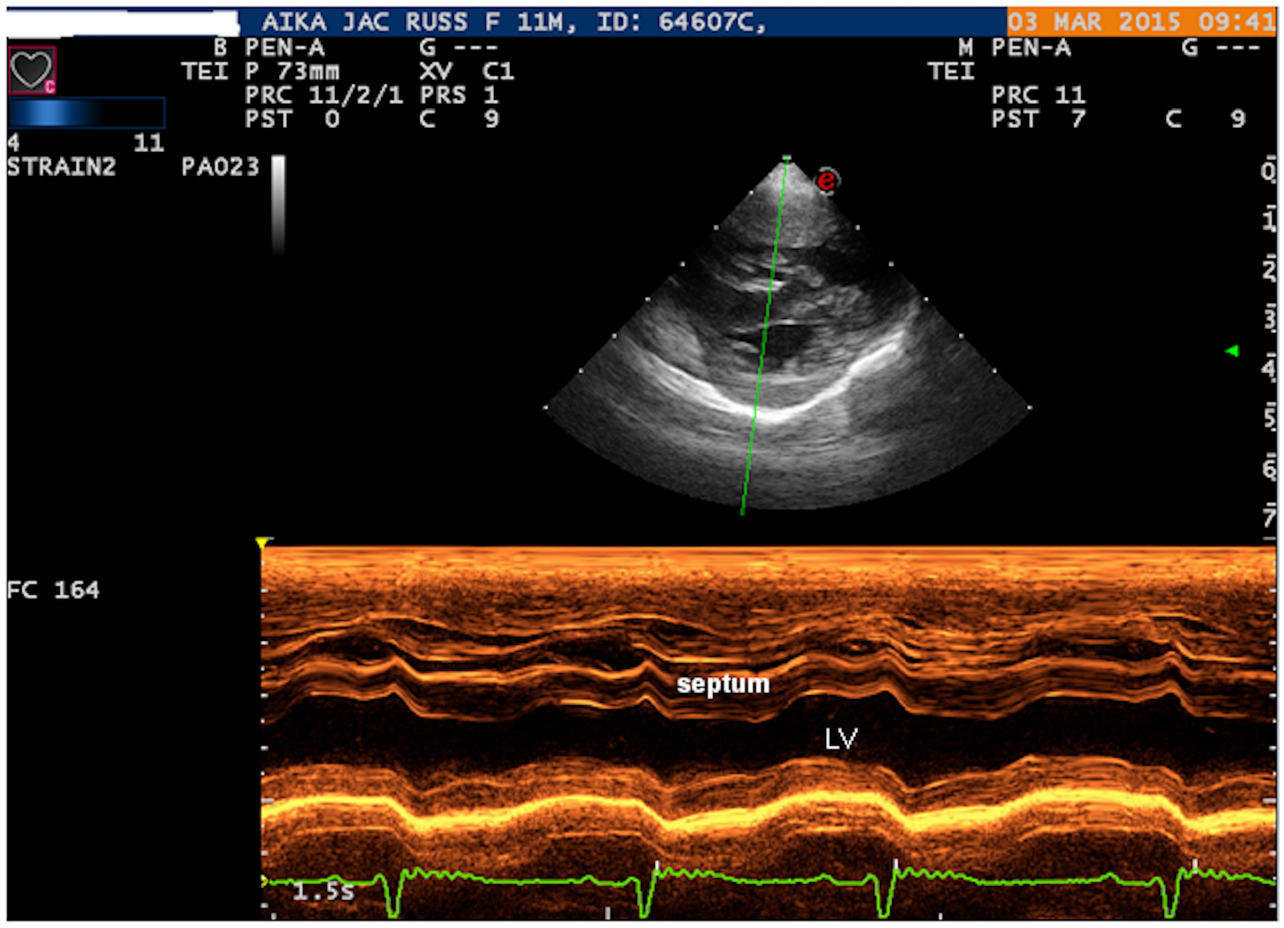
Right parasternal short axis view: M-MODE showing the right ventricular wall hypertrophy and systolic flattening of the interventricular septum. LV, left ventricle.

Two-dimensional echocardiography (Philips Ultrasound EPIQ Elite, Philips S.p.A.) demonstrated a large, isolated mid-muscular ventricular septal defect (7 mm) (Figure 1, Supplementary Videos S1, S2). The right parasternal short axis view, at the level of the left ventricular papillary muscles, showed right ventricular wall hypertrophy (1 cm) and systolic flattening of the interventricular septum, without pulmonary stenosis (Figure 2). Color-flow Doppler revealed bidirectional shunting through the large VSD (Figure 1). The absence of pulmonary or tricuspid regurgitation precluded estimation of the blood pressure within the pulmonary circulation and the owners did not consent to the suggested pulmonary catheterization to directly measure the pulmonary artery pressure. No other congenital heart diseases, associated with VSD, were found.

A blood sample was collected for CBC and the only alteration was a hematocrit (HCT) of 68.5% (reference range, 37%−55%); another blood sample was collected to perform serum biochemistry, but the serum obtained was insufficient and highly hemolytic, and the serologic test for *Dirophilaria immitis* (negative).

A diagnosis of VSD with ES was made and therapy with sildenafil citrate (0.5 mg/Kg twice daily) was initiated (20). Over the next 3 months (months 2, 3, and 4) the dog's clinical condition and hematocrit remained stable and her appetite improved. At month 5 (5.4 Kg body weight), the HCT was 73%, a phlebotomy was performed (10 mL/Kg of blood was drawn at each phlebotomy) and sildenafil dosage was increased (1 mg/Kg twice a day) because owners reported increased duration of polypnea after physical activity (15). Over the next months, cyanotic MM and easy fatigability with moderate physical activity persisted, and numerous episodes of syncope occurred (the dog was active and playing with a ball). Phlebotomy was repeated every month and the sildenafil dosage was progressively increased. At month 12, the phlebotomy was repeated and the sildenafil dose reached 1.5 mg/Kg three times daily (21). The subsequent physical examination (month 16) revealed an improvement in the clinical condition and a decrease in syncopal episodes. Systolic blood pressure was 120 mmHg, HR was 140 bpm, HCT was 70% and the echocardiographic evaluation was unchanged (6.2 Kg body weight).

From month 17 to month 73, regular follow-ups were carried out once a year and the clinical conditions of dog remained stable without the need for phlebotomy, with sporadic episodes of syncope during sustained physical activity.

The owner contacted us in April 2021 (month 74) because the dog had been showing dysorexia for 2 weeks, 2 episodes of vomiting, soft feces with mucus and decreasing activity (6.5 Kg body weight). Echocardiography, abdominal ultrasound, chest radiograph evaluation, venous blood gas analysis (vBGA), CBC and biochemical profile were performed (Table 1). The HCT was 80% and the vBGA revealed metabolic acidosis and an increase in bilirubin and transaminases. A phlebotomy was performed and the sildenafil dosage was increased from 1.5 to 2 mg/Kg in the morning and evening (5.5 mg/Kg daily). After 6 h the dog was more active, ate with appetite and was discharged after 12 h of hospitalization for observation. At the follow up conducted in June 2021 (month 76), the owner reported an improvement in physical activity and normalization of appetite with no further episodes of vomiting or soft feces; no episodes of syncope were reported.

**Table 1:** Results of hematologic, biochemical, and venous blood gas parameters performed.

**Variable**	**Result at month 74**	**Result at month 76**	**Result before surgery**	**Result after 11 days from surgery**	**Institutional reference values**
pH	7.36		7.47		7.34–7.38
PCO_2_ (mmHg)	**26**		21.9		40–46 mmHg
SBE (mmol/L)	**−10.2**		−7.9		−2-+2 mmol/L
Lactated (mmol/L)	1.1		1.7		0.5–2 mmol/L
Sodium (mmol/L)	149		146		140–150 mmol/L
Potassium (mmol/L)	4		4.5		3.9–4.9 mmol/L
Chloride (mmol/L)	**123**		118		109–120 mmol/L
Ionized calcium (mmol/L)	1.26		1,39		1.25–1.5 mmol/L
Urea (mg/dL)	49.9	43.63	32.35	48.18	17.2–59.2 (mg/dL)
Glucose (mg/dL)	**56**	84.96	96.25	100.2	71–126 (mg/dL)
GGT (UI)	**80.14**	**11.42**	9.77	11.44	2–9 (UI)
Creatinin (mg/dL)	0.99		0.32	1.33	0.5–1.8 (mg/dL)
ALP (UI)	**1565.69**	104.78	729.2	361.3	7.2–183.2 (UI)
Bilirubin (mg/dL)	**1.02**	0.43		0.48	< 0.450 (mg/dL)
ALT (UI)	**352.84**	**208.58**	86.31	103.28	18.1–96.8 (UI)
Albumin (g/dL)	3.16	3.23	2.4	2.77	2.8–4 (g/dL)
Total protein (g/dL)	6.09	7 g/dL	6.75	7 g/dL	5.5–7.2 (g/dL)
WBC (cells/μL)	10.7x10E03		**22.9x10E03**		5.2–17.9 (cells/μL)
RBC (cells/μL)	**12.8x10E06**		8.19x10E06		5.4–8.6 (cells/μL)
HCT (%)	**81.5**	**65%**	**65%**		37.5–58.3 (%)
PLT (cells/μL)	133 x10E03		158 x10E03		127.7–543 (cells/μL)

pCO_2_, partial pressure of carbon dioxide; SBE, base excess (ABL90 FLEX - Radiometer Medical ApS, Åkandevej 21, Brønshøj, Denmark); GGT, gamma glutamyl transferase; ALP, alkaline phosphatase; ALT, alanine transferase (ILAB 300 plus, Clinical Chemistry System, Instrumentation Laboratories); WBC, white blood cell count; RBC, red blood cell count; HCT, hematocrit; PLT, platelet (ADVIA 120 Hematology, Siemens Healthcare Diagnostics). Results indicated in bold are out of institutional reference values.

In June 2022 (month 88), the owners observed increasing fatigue during walking (decrease in walking distance) and on the physical examination the MM were cyanotic, the HCT was 74% and the dog had lipothymia; the sildenafil dosage was increased to 2 mg/Kg 3 times a day.

In July 2023 (month 101), the dog (6.7 Kg) was conducted to the hospital for sanguineous vulvar discharge, during the past 10 days. Physical activity was normal, body temperature 38.4°C, HR 120 bpm, 24 breaths per minute, CRT < 2 seconds, normal metatarsal pulse, 120 mmHg blood pressure with Doppler, 98% SpO2, 70% HCT and 7 g/dL total protein. The following analyses were performed: CBC, biochemical panel, vBGA (all parameters were in the reference ranges), abdominal ultrasound, determination of serum progesterone levels, vaginal cytology, swab, and culture. An open pyometra was diagnosed and treatment with marbofloxacin (2 mg/Kg daily, based on culture results) and aglepristone (10 mg/Kg SC daily, repeated the next day and after 7 days) was started.

The dog was brought to the hospital in November (month 105) for anorexia and obtundation (body weight 6.4 Kg). The clinical examination revealed: 39°C body temperature 220 bpm HR (sinus ECG), 36 breaths per minute, CRT ≥3 seconds, weak metatarsal pulse, 110 mmHg blood pressure, 95% SpO_2_, 60% HCT and 7 g/dL total protein. The ultrasound evaluation of the abdomen showed that the body and horns of the uterus were filled with fluid and had increased significantly in diameter (maximum 8 cm); a small amount of free fluid was also present. The dog was hospitalized and a CBC, biochemistry, vBGA and echocardiography were performed (unremarkable results) (Table 1). After application of intravenous catheter in the left cephalic vein, fluid therapy with Ringer lactate (bolus of 5 ml/Kg for 10 min, followed by maintenance plus replacement, considering 7% of dehydration), methadone (0.2 mg/Kg), maropitant (1 mg/Kg daily) and marbofloxacin (2 mg/Kg daily) were administered (sildenafil, 2 mg/Kg 3 times a day; it was administered in the morning by the owners); surgery was performed after a few hours.

After preoxygenation, a bolus of remifentanil (0.3 mcg/Kg IV) was administered, followed by an infusion of propofol (40 mg/Kg/h IV) to induce general anesthesia. The dog was intubated and a mixture of medical air and oxygen with a fraction of inspired oxygen of 0.7 was delivered using a rebreathing system. Pressure-support ventilation was applied to maintain values of end tidal carbon dioxide between 35–40 mmHg. A volume of 5 mL/Kg/h of lactated Ringer's solution was administered intravenously. The left dorsal pedal artery was cannulated to invasive monitoring of blood pressure and anesthesia was maintained with propofol (20–18 mg/Kg/h IV) and remifentanil (0.2–0.5 mcg/Kg/min). Cardiovascular and respirator parameters, SpO_2_ and esophageal temperature were monitored intraoperatively. Splash block on both ovarian pedicles was performed with lidocaine (4 mg/Kg). Fifteen min after induction, hypotension was detected (MAP 52 mmHg), an arterial blood sample was collected from the arterial line and dobutamine was administered at 3 mcg/Kg/min (Table 2). Five min later MAP was 65 mmHg: a bolus of lactated Ringer's solution (3 ml/Kg IV) was provided and the infusion of dobutamine was increased to 5 mcg/Kg/min. Blood pressure was restored (MAP 90 mmHg) and maintained between 65–95 mmHg. Heart rate ranged from 100 to 140 bpm with sinus rhythm. The anesthesia lasted 55 min. Ovariohysterectomy was performed through midline celiotomy following standard internal procedures. The uterus was greatly enlarged, with purulent content, and the ovaries showed recent corpora lutea.

**Table 2:** Results of arterial blood gas parameters performed during hospitalization for pyometra.

**Variable**	**Sample obtained during anesthesia (FiO_2_ = 70%)**	**Sample obtained during awakening in the ICU (FiO_2_ = 21%)**	**Sample obtained in the late night in ICU (FiO_2_ = 21%)**	**Institutional reference values**
pH	**7.22**	7.38	**7.43**	7.34–7.38
PCO_2_ (mmHg)	**52.5**	**34.2**	**27.4**	40–46 mmHg
PaO_2_ (mmHg)	**48.7**	**24.7**	**35.8**	80–105 mmHg
SaO_2_ (%)	**66.4**	**31.6**	**62.3**	≥98%
PaO_2_/FiO_2_	**69.6**	**117**	**170**	>400
(A–a) gradient		**79.5**	**76**	< 15
SBE (mmol/L)	**−6**	**−5**	**−6.3**	– 3 mmol/L
Lactated (mmol/L)	0.9	1.2	0.8	0.5–2 mmol/L
Sodium (mmol/L)	148	149	149	140–150 mmol/L
Potassium (mmol/L)	4.3	4.7	4.6	3.9–4.9 mmol/L
Chloride (mmol/L)	120	119	**122**	109–120 mmol/L
Ionized calcium (mmol/L)	1.54	1.44	1.40	1.25–1.5 mmol/L

FiO_2_, fraction of inspired oxygen; PCO_2_, partial pressure of carbon dioxide; PaO_2_, partial pressure of arterial oxygen; SaO_2_, oxygen saturation level; SBE, base excess; Results indicated in bold are out of institutional reference values. The emogas analyzer is an ABL90 FLEX (Radiometer Medical ApS, Åkandevej 21, Brønshøj, Denmark).

On awakening from anesthesia, an arterial blood sample was taken from the arterial line and a nasal probe was applied for oxygen administration. Serial arterial BGA were reported in Table 2. Methadone was prescribed as needed and meloxicam (0.1 mg/Kg daily) was added the next day. In the postoperative period, the dog was hemodynamically stable, started eating the day after surgery and had no polypnea or dyspnea at rest, but slight increase in the respiratory effort (min 24 and max 36 breaths/min); she tolerated short walks for physiological needs, but subsequently had marked polypnea and cyanosis of the MM. In the morning of the third day after surgery, the RR at rest was 24 breaths per minute and SpO_2_ was 93% without oxygen (the arterial line was removed due to clots; an unsuccessful attempt was made to collect an arterial blood sample while the dog was awake). The dog remained stable without oxygen therapy and was discharged from the hospital in the late afternoon, with the prescription to keep at rest, check the RR and MM color, and continue administration of marbofloxacin and meloxicam for 2 and 4 days respectively.

A telephone follow-up was performed on the 4^th^ and 8^th^ day after discharge. The owners reported that the dog did not move much during the first few days and easily developed polypnea, but from the 4^th^ day, she started to become more active and bark. When she was in the garden for a brief walk, she frequently needed to rest, breathing laboriously. At the second call, her appetite was voracious again, physical activity had improved (she no longer needed to rest) and polypnea had also decreased. Eleven days after surgery, the dog was active, the appetite was normal and after physical activity the polypnea lasted slightly longer than usual. The examination was normal and the clinical parameters were as follows: 140 bpm of HR, body temperature 38°C, SpO_2_ 96%, normal metatarsal pulse, polypnea and MM slightly congested. A blood sample was collected to check the biochemistry and the metal staples were removed (Table 1).

## Discussion

The clinical case described the long-term treatment with sildenafil in a dog with heart failure secondary to VSD and pulmonary hypertension (ACVIM classification 1d1) (12, 22). The therapy allowed the dog a good quality of life with moderate physical activity (running, playing, and barking), although recovery from polypnea takes longer than in a healthy dog. No systemic hypotension, change in the flow characteristics of the bidirectional shunt, or other adverse effects associated with sildenafil therapy were observed, but the long-term and high-dose therapy is quite expensive.

The emergency treatment and the recovery from pyometra, a pathology that affected the dog last year, at the age of 9 years and 7 months, was also described. An ovariohysterectomy was performed to treat the pyometra and the major problem encountered during anesthesia was hypotension. In this type of patient, the drop in systemic blood pressure leads to an increase in the shunt volume and a greater proportion of non-oxygenated blood reaches the systemic circulation, resulting in a marked decrease in SpO_2_. The dog responded well to the infusion of dobutamine (3–5 mcg/Kg/min) and systemic blood pressure was promptly restored.

In the post-operative period, the major concern in the management of the dog was the severe hypoxemia (Table 2). The dog had tolerated the hypoxia well, with no particular clinical manifestations at rest (Supplementary Video S3) and had started eating again 12 h after surgery. After anesthesia, arterial gas analyses showed severe hypoxemia (with normal lactate), secondary to venous admixture, and a decrease in pCO_2_. Pre-operative biochemistry showed elevated transaminases, which could indicate a liver damage secondary to systemic disease (uterine infection and hypoperfusion); the increase in gamma glutamyl transferase and alanine transferase after surgery suggested that the hepatic injury was likely ongoing due to low arterial oxygen pressure. Liver and gut are reported as more vulnerable to hypoxic insult than other organs, indeed liver damage had already been noted at month 74, when the dog was hospitalized for hyperviscosity syndrome, manifested by gastrointestinal signs (23).

Across large septal defects, a great amount of blood shifts from the left to the right ventricle due to pressure difference between systemic and pulmonary circulation (systolic pressure in left ventricle is almost 120 mmHg compare to 20 mmHg in the right ventricle) (24). When pressure in the right site of the heart increases and approaches the pressure in the left side, for example secondary to pulmonary stenosis or pulmonary hypertension, the shunt can reverse from right to left. Pulmonary hypertension develops in such patients as a result of abnormal changes in the pulmonary vasculature, due also to the large amount of blood reaching the pulmonary arteries. In human patients with VSD or PDA and large defects, PH develops in the 1^st^ years of life (13, 25). In dogs, reversed shunting usually develops earlier than in humans, often before 6 months of age (1). The increase in pulmonary blood flow and pressure exerts shear and circumferential stresses on the endothelium and, acting chronically, can lead to endothelial dysfunction (26). The decrease in nitric oxide and prostacyclin production and the increased expression of vasoconstrictors (e.g., thromboxane A_2_ and endothelin) increase vascular tone and promote vascular remodeling (27). Initial morphological changes such as medial hypertrophy and intimal proliferation may be reversible, but irreversible lesions (plexiform lesions and arteritis) occur as the disease progresses (28, 29). In reversed shunting, oxygenated and non-oxygenated blood are mixed, leading to persistent hypoxia in the systemic circulation, which triggers the production of erythropoietin by the kidney and the development of secondary erythrocytosis and hyperviscosity (15).

Patients with ES may present with dyspnea, fatigue, or syncope, and subtle neurologic abnormalities due to blood hyperviscosity (30). These types of patients have a very low intensity murmur, because the higher viscosity and low flow velocity of the blood reduces flow turbulence (13). Repeated phlebotomy is performed to relieve the symptoms of hyperviscosity, but excessive blood loss can lead to iron deficiency and microcytosis, which exacerbates the hyperviscosity (30). In dogs affected by right-to-left shunting and polycythemia, morbidity and mortality are generally thought to be linked to the effects of chronic hypoxemia and erythrocytosis. Regular phlebotomies and PDE-5 inhibitors have been recommended to help manage polycythemia and clinical signs (1, 12, 15).

In veterinary medicine, there are only a few case series and one retrospective study describing the long-term management of dogs with polycythemia secondary to PDA with right-to-left shunt. Côté et al. reported successful treatment outcomes with phlebotomy performed “as needed” in 4 dogs, depending on clinical findings (18). Instead, Moore et al. treated 4 dogs with hydroxyurea for several months, to alleviate clinical signs secondary to erythrocytosis (19). Finally, Nakamura et al. administered sildenafil therapy (0.5 mg/Kg twice daily) in 5 dogs with ES secondary to a reversed PDA, and found an improvement in secondary erythrocytosis and clinical signs after 3 months (20). The retrospective study observed 35 dogs with bidirectional and right-to-left PDA and showed a median survival time of 1 years and 7 months (range from 1 day to 9 years and 9 months), which increased to 5 years considering the dogs without cardiac failure (13). Moreover, dogs lived longer when treated with sildenafil at presentation (5 years vs. 8 months without sildenafil, *P* = 0.03) (13).

In humans, the administration of selective pulmonary vasodilators (e.g., sildenafil or bosentan) also alleviates the symptoms of the syndrome, improving quality of life, and prolongs survival (31, 32). However, these drugs can also reduce systemic pressure, leading to an increase in right-to-left shunt and decreased SpO_2_ (33, 34). Sildenafil is a PDE-5 inhibitor that promotes nitric oxide-cyclic guanosine monophosphate (cGMP)-induced vasodilation by inhibiting the degradation of cGMP and has been associated with antiproliferative effects on pulmonary vascular smooth muscle cells (35, 36). In dogs, sildenafil has a short half-life, ideally requiring an 8-h dosing, and rectal administration may be considered if oral administration is not feasible (37, 38).

Surprisingly, no echocardiographic parameters were available to estimate pulmonary pressure, although careful attention was paid to the presence of pulmonary and tricuspid regurgitation for at every echocardiographic examination performed during the dog's life. Therefore, HCT and clinical signs such as HR, RR, syncope, and physical activity, were used to monitor the dog's response to sildenafil citrate therapy. In human medicine, the determination of exercise capacity plays an important role in the evaluation of patients with PH and is a recognized surrogate that correlates with survival, functional class, hemodynamics, echocardiographic parameters, biomarkers, and health-related quality of life (39–41).

In conclusion, although this dog has a congenital heart defect that cannot be treated with surgery, medical treatment has allowed her a good quality and long life. Dog has also undergone a surgery, which was successful, with good and quick recovery.

Chronic therapy was well tolerated and no side effects were reported. In addition, treatment with sildenafil has reduced symptoms (syncope and fatigue) and dramatically reduced the need of phlebotomy which, in the long term, would have been a source of stress for the dog and the owners.

Periodic follow-ups to evaluate the symptoms and the haematocrit value, are important to modulate therapy during the life.

## Data Availability

The original contributions presented in the study are included in the article/Supplementary material, further inquiries can be directed to the corresponding author.
