# Supplementary material for: Case report: Eisenmenger syndrome in a dog with ventricular septal defect: long term management and complications
Source: Front Vet Sci. 2024 Nov 1;11:1393919. doi: 10.3389/fvets.2024.1393919 (PMC11565600; doi:10.3389/fvets.2024.1393919)
Supplement: Supplementary file 1 [file Table_1.docx]

Supplementary Material

Eisenmenger syndrome in a dog with ventricular septal defect: long term management and complications

Barbara Bruno^*^; Paolo Savarino, Claudio Bussadori, Andrea De Giovanni, Elena Lardone, Alessia Bertero, Alberto Tarducci

*** Correspondence:** Barbara Bruno: [barbara.bruno@unito.it](mailto:barbara.bruno@unito.it)

**Table S1:** Results of ultrasonographic evaluation of uterus, vaginal cytology and cultural analysis of the vaginal swab, performed at month 101.

| Ultrasound evaluation | Anechoic content was detected in the uterus lumen with maximum repletion in the right horn (1.4 cm). Reduction in wall tension currently with an average thickness of approximately 2 mm (compared to 1.25 in the previous scan). Suspected lesion identified close to the junction between the right and left horn appears organized in a particularly reactive endometrial formation with small intraluminal fluid collection. | | |
| --- | --- | --- | --- |
| Vaginal cytology | Cytology compatible with diestrus with abundant presence of erythrocytes and rare neutrophils | | |
| Cultural and susceptibility testing | Escherichia Coli | | |
|  |  | Resistant | Sensitive |
|  | Amoxicillin | x |  |
|  | Amoxicillin/  Clavulanic acid | x |  |
|  | Ampicillin | x |  |
|  | Enrofloxacin |  | x |
|  | Marbofloxacin |  | x |
|  | Cefalexin |  | x |
|  | Cefepime |  | x |
|  | Cefovecin |  | x |
|  | Ceftriaxone |  | x |
|  | Amikacin |  | x |
|  | Gentamicinte |  | x |
|  | Tetracycline |  | x |
|  | Trimethoprim/Sulfamethoxazole |  | x |

**Video S1.** Right parasternal short axis view and color-flow Doppler of the bidirectional shunting through the ventricular septal defect.

**Video S2.** Left parasternal apical four chambers view of the ventricular septal defect.

**Video S3.** The dog at rest in the cage, the day after surgery.
